# Improving Self-Care of Informal Caregivers of Adults With Frontotemporal Degeneration

**DOI:** 10.1093/geroni/igab046.1453

**Published:** 2021-12-17

**Authors:** Lauren Massimo, Michelle Sharkey, Lauren Fisher

**Affiliations:** University of Pennsylvania, Philadelphia, Pennsylvania, United States

## Abstract

Frontotemporal degeneration (FTD) is a common cause of young-onset dementia that results in progressive deterioration in executive functioning and social comportment. A tremendous burden is placed on young caregivers, typically spouses, who often sacrifice their own self-care needs in order to manage the cognitive decline and subsequent functional impairments of their loved one, contributing to extraordinarily high levels of stress and depression in caregivers of individuals with FTD. Very few interventions have been tested specifically in FTD caregivers, and those that exist have generally focused on education around patient behavior management. In this session, we will discuss how we adapted the iCare4Me study, originally designed for heart failure caregivers, for caregivers of persons with FTD and we will share initial findings from iCare4Me for FTD, a randomized controlled trial which evaluates the efficacy of a virtual health coach intervention aimed at increasing self-care behaviors and reducing stress in FTD caregivers.

